# Hepatitis C Virus Infection Cycle-Specific MicroRNA Profiling Reveals Stage-Specific miR-4423-3p Targets RIG-I to Facilitate Infection

**DOI:** 10.3389/fcimb.2022.851917

**Published:** 2022-03-24

**Authors:** Xijing Qian, Bingan Wu, Chen Xu, Zhongtian Qi

**Affiliations:** ^1^ Department of Microbiology, Faculty of Naval Medicine, Naval Medical University, Shanghai, China; ^2^ Spine Center, Department of Orthopedics, Shanghai Changzheng Hospital Affiliated to Naval Medical University, Shanghai, China

**Keywords:** hepatitis C virus, microRNA, miR-4423-3p, RIG-I, small RNA sequencing

## Abstract

Hepatitis C virus (HCV) infection is one of the main causes of chronic liver diseases, the disorders of which involve multiple pathological processes and elements including host factors such as non-coding small RNAs. Although several genes have been reported to be correlated with HCV infection, the potential regulatory network has not been deciphered clearly. By small RNA sequencing, we clarified the expression profile of microRNAs (miRNAs) in HCV-infected Huh7 and Huh7.5.1 cells and identified 6 dysregulated miRNAs with the same expression trend and 32 dysregulated miRNAs with different expression trends during different stages of HCV life cycle. By looking into each infection stage, we found that 6 miRNAs were entry stage specific, 4 miRNAs were replication stage specific, and 1 miRNA was related to the transmission stage. Moreover, due to the fact that Huh7.5.1 cells have a retinoic acid-inducible gene 1 (RIG-I) mutation which causes reduced production of interferons (IFNs), we here focused on the miRNAs of different trends to decipher the RIG-I/IFN specific miRNAs. Among them, miR-4423-3p showed a significant promotive effect on HCV infection by suppressing RIG-I/IFN pathway through direct binding to RIG-I mRNA. Together, the results displayed novel insights into the miRNA regulatory networks in HCV infection and progression, thus providing a prosperous perspective into the establishment of novel therapeutic and diagnostic targets of the disease.

## Introduction

Hepatitis C virus (HCV) infection affects more than 71 million people worldwide and is one of the major causes of chronic liver diseases, the end stage of which will progress into multi-organ failures (WHO, [[NoYear]]; Manns et al., 2017; Dore and Bajis, 2021). The abuse of drug injection epidemically results in the increase of diagnosed HCV patients, especially in young adults (Suryaprasad et al., 2014). There are still no effective ways to prevent and treat the occult HCV infection (Wroblewska et al., 2021). Although the direct-acting antivirals (DAAs) which are developed and updated since 2011 revolutionarily elevate the treatment efficacy in HCV patients, they are limited by restricted genotype activity, drug–drug interactions, significant adverse reactions, and high costs (Li and Chung, 2019; Gao et al., 2021; Lazarus et al., 2021). Besides, no optimal vaccines are available for HCV till now.

The replicative cycle of HCV involves multiple viral and host factors to participate in including non-coding RNAs (Esteller, 2011). There are studies concerning various genes which are closely associated with HCV infection (Peng et al., 2009; Qian et al., 2016; Qian et al., 2020). However, no specific genes or their exact regulated mechanisms in HCV pathogenesis are systemically mentioned.

MicroRNAs (miRNAs) are non-coding small RNAs of about 20 nucleotides long which participate in diverse physiological and pathological processes. They function by binding to the complementary regions of targeted mRNAs, resulting in the degradation of mRNAs or the repression of their translation (Benfey, 2003). A series of miRNAs were identified to be up or downregulated during HCV infection (Li et al., 2016). For example, the expression of miR-483-5p and miR-215 was found to be increased while miR-122 be decreased in the serum of patients with chronic hepatitis (Sadri Nahand et al., 2019; Kunden et al., 2020). However, miRNAs have a complicated regulatory system. One miRNA is capable of targeting multiple mRNAs, and one mRNA can also be suppressed by various miRNAs (Krutzfeldt et al., 2005). Therefore, exploring a global miRNA regulatory network during HCV infection is of great necessity and urgency, depicting the real state of miRNA functions in the disease.

During HCV infection, host immune responses are key factors to affect disease progression and outcomes. Huh7.5.1 cells have a point mutation in retinoic acid-inducible gene 1 (RIG-I) which recognizes viral RNA to activate type I IFNs synthesis (Sumpter et al., 2005), and thus being more susceptible to HCV infection than Huh 7 cells due to the weak antiviral responses (Zhong et al., 2005). Therefore, we focused on the differed expression profiles in Huh7 and Huh7.5.1 cells, trying to figure out relevant miRNAs concerning key elements in RIG-I pathway.

Here we utilized high-throughput sequencing and bioinformatics technology to make an integrative analysis of the regulatory miRNA networks in HCV-infected cells compared with the noninfected cells. The identified differentially expressed miRNAs were further clarified and investigated for their potential functions. Furthermore, the specific miRNA/mRNA interaction of miR-4423-3p with RIG-I was confirmed which was closely related to IFN production and responsible for host antiviral responses. Taken together, our data indicated that miRNA regulatory networks which were comprised of the specific miRNAs expression profiling and their predicted functioning genes played important roles in HCV infection, providing potential optimal therapeutic and diagnostic targets for the disease.

## Materials and Methods

### Cells and Reagents

Human hepatoma Huh7 cells from the National Collection of Authenticated Cell Cultures (Chinese Academy of Sciences, Shanghai, China) and Huh7.5.1 cells (a gift from professor C. Rice) were cultured in Dulbecco’s modified Eagle’s medium (DMEM) supplemented with 10% fetal bovine serum (FBS), 100 U/ml streptomycin and penicillin, 1× non-essential amino acids (NAAs) and 2 mM L-glutamine (GIBCO-Invitrogen, Carlsbad, CA, USA). The cells were incubated accordingly at 37°C with 5% CO_2_. Anti-HCV core and anti-RIG-I monoclonal antibodies were purchased from Abcam (Toronto, Ontario, Canada). Alexa 488-and horseradish peroxidase (HRP) conjugated anti-mouse IgG and 4’,6-Diamidino-2-phenylindole (DAPI) (Invitrogen, Carlsbad, CA, USA) were used in the study. Amlexanox (HY-B0713) was obtained from MedChemExpress (Shanghai, China).

### Virus and Infection Assay

Cell culture-derived hepatitis C virus (HCVcc) was produced using the plasmid of Japanese fulminant hepatitis type 1 (JFH-1) genome, kindly provided by T. Wakita (National Institute of Infectious Diseases, Tokyo, Japan) as described previously (Zhong et al., 2005). Briefly, the plasmid was linearized and transcribed *in vitro* using a MEGAscript kit (Promega, USA). As for the infection assay, Huh7 or Huh7.5.1 cells were incubated with HCVcc of JFH-1 for 6 h at 37°C. The supernatants were then discarded and re-supplemented with fresh medium. The cellular infection was then detected 48 h post virus inoculation by immunofluorescent (IF) assay using anti-HCV core monoclonal antibody or real-time quantitative polymerase chain reaction (RT-qPCR).

### High-Throughput Small RNA Sequencing and Bioinformatics Analysis

Huh7 and Huh7.5.1 cells were infected with HCVcc of JFH-1 and total RNA was then extracted from the respective cells at indicated time points (0, 6, 24, and 48 h) after infection using TRIzol reagent (Invitrogen, Carlsbad, USA) accordingly to perform the transcriptome sequencing. As for the small RNA sequencing, a total RNA of 10 mg for each sample was acquired to prepare the small RNA cDNA library according to a previous study with some appropriate adjustments (Xu et al., 2016; Qian et al., 2020). The RNA libraries of specific strands were formed by utilizing a TruSeq Small RNA Sample Prep kit (Illumina, San Diego, United States). Briefly speaking, small RNA fragments of 15 to 100 nt were isolated, purified, and ligated to approximately 30 to 50 adaptors. They were subsequently reverse transcribed to cDNA, followed by amplification using polymerase chain reaction (PCR). The entire libraries were examined by gel electrophoresis, and corresponding bands related to miRNA insertion were cut off and eluted. The small RNA libraries were then purified with repeated ethanol precipitation and washing, and quantified for the subsequent sequencing analysis by an Illumina HiSeq™ 2500 analyzer (Illumina, San Diego, United States) following the instructions of the manufacturer. The entire profiling assays were carried out with the help of the Shanghai NovelBio Bio-Pharm Technology Co., Ltd.

### Immunofluorescence Assay

At 48 h post-infection, Huh7 or Huh7.5.1 cells were fixed by cold methanal and incubated at −20°C for 30 min, followed by blockage with 3% bovine serum albumin at room temperature for 2 h. HCV infection was then tested by anti-HCV core monoclonal antibody and corresponding secondary antibody. The infection rate was analyzed by a fluorescence microscope cell imaging system with the mean ratio of positive cells calculated with six random fields in the well.

### Real-Time Quantitative Polymerase Chain Reaction

Total cellular RNA was extracted using TRIzol reagent (Invitrogen, USA) according to the instructions of the manufacturer, and quantified at 260 nm with a spectrophotometer. The RNA was then reverse-transcribed with a PrimeScript™ RT Master Mix kit (Takara, Japan). RT-qPCR was performed using TB Green Premix^®^ Ex Taq™ (Takara, Japan) with a Step One real-time PCR system (Applied Biosystems, USA). GAPDH was utilized as mRNAs endogenous control. The sequences of primers used in the study are listed in **Supplementary Table 1**. For miRNA detection, we used commercially available Taqman™ primers (Thermo Fisher Scientific, USA) to assess the expression of miRNAs.

### MiRNA Mimics and Inhibitor Synthesis and Transfection

To determine the role of miR-4423-3p on viral infection, the mimics and inhibitors (antagomir) of miR-4423-3p were purchased from the GenePharma Corporation (Shanghai, China) and transfected into Huh7 or Huh7.5.1 cells by FuGENE HD transfection reagent (Promega, USA) according to the instructions of the manufacturer. The small interfering RNAs (siRNAs) for RIG-I knockdown were synthesized by the GenePharma Corporation, and the sequences for all constructs are shown in **Supplementary Table 1**.

Transfection was conducted according to a previous report (Qian et al., 2016). Briefly, miRNA mimics, antagomir, overexpression plasmid, or siRNAs were incubated with transfection reagent in a serum-free culture medium before they were transferred to the supernatant of the cells. Cells were then incubated with the transfection complexes for 24 h before the medium change.

### Generation of HCV Pseudoparticles (HCVpp) and Entry Assay

HCV pseudoparticle (HCVpp) was produced according to a previous study (Tong et al., 2011). Briefly, HEK 293T cells were transfected with plasmids encoding viral envelope protein (H77 strain, kindly provided by Professor F.L. Cosset), Gag/Pol, Rev, and pLenti6 transferring vector by Lipofectamine™ 2000 (Invitrogen). The culture medium was refreshed 6 h after transfection, and cellular supernatant was collected and filtered 48 h post-transfection.

The entry efficiency was detected according to a previous study (Bartosch et al., 2003). Huh7 cells were incubated with HCVpp for 6 h before the medium change. The HCV entry was evaluated 72 h post-incubation by counting the positive cells in randomly six fields of the well under a fluorescence microscope cell imaging system.

### Dual-Luciferase Reporter Assay

The wild type or mutated 3’UTR of RIG-I or IFN-γ reporter constructions were synthesized and subcloned into pMIR-REPORT vector (Promega, USA) by the GeneChem Corp (Shanghai, China). Huh7 cells in 96-well plates were co-transfected with pMIR-REPORT vector (50 ng wild type or mutated plasmid) and miRNA mimics or scramble control using Lipofectamine™ 2000. Approximately 48 h post-transfection, the Dual-Luciferase Reporter Assay System (Promega, USA) was utilized to test the luciferase activity according to a previous study (Liu et al., 2020). The intensity was normalized by Firefly luciferase.

## Results

### HCV Infection Cycle-Specific MicroRNA Profiling Reveals Distinct Expression Patterns Using Different Cell Lines

**Figure 1:**
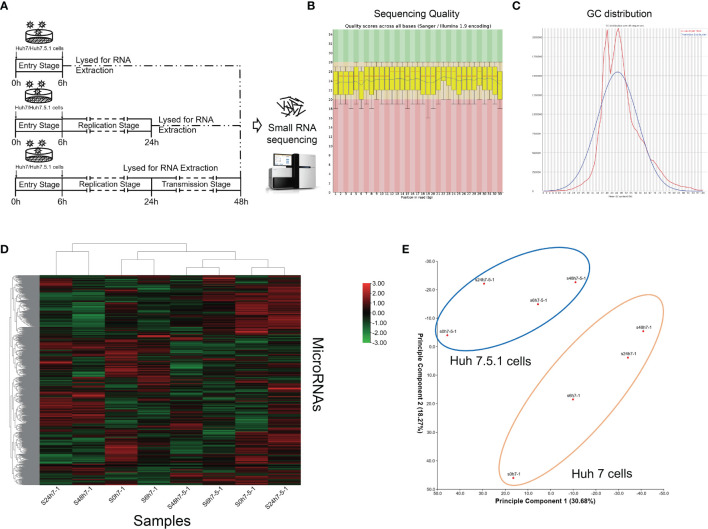
miRNA expression profiles of high-throughput sequencing analysis during HCV infection. **(A)** Schematic graph of small RNA sequencing. **(B)** Representative images of sequencing quality scores of the samples. **(C)** GC distribution of the sequencing data. **(D)** Hierarchical clustering of the sequenced samples. Note that samples of the same cell line were clustered together, and the time point was chosen according to miRNA deviation degree during HCV infection. The red color represented upregulated miRNAs, and green color represented downregulated miRNAs. **(E)** Principal component analysis showing the distribution of samples. Note that the samples of same cell line were circled together.

To gain insights into the global microRNA (miRNA) expression during different stages in the whole life cycle of HCV, we utilized Huh7 and Huh7.5.1 cells and infected them with HCV for different periods of time (6, 24, and 48 h). The cells were then lysed at indicated time points and subjected to high-throughput miRNA sequencing analysis (**Figure 1A**). Clean reads were generated and mapped with annotated mature miRNAs in the miRbase database (version 20) (**Figures 1B, C**). Hierarchical cluster analysis was then carried out using Euclidean distance similarity metric to compare and examine the similarity among these samples, and it was found that substantial differences existed in miRNA expression during different stages of HCV infection (**Figure 1D**). Through principal component analysis, it is shown that the miRNA expression patterns were distinct not only between different infection stages, but also between different cell lines (**Figure 1E**).

### Comparative MiRNA Expression Analysis During Infection Revealed HCV-Specific MiRNA Signatures

**Figure 2:**
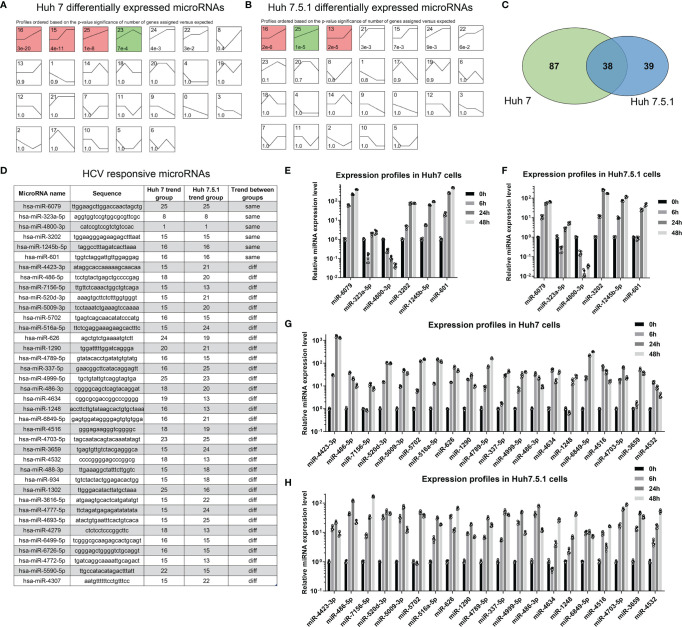
miRNA signatures were different between Huh7 and Huh7.5.1 cells. **(A, B)** miRNA expression changes were categorized using series cluster method, and was classified into 26 different expression pattern groups in Huh7 and Huh7.5.1 cells. **(C)** Venn plot showing the differentially expressed miRNAs in both cell lines. **(D)** The sequences of HCV responsive miRNAs in Huh7 or Huh7.5.1 cells were illustrated, and their change profiles were identified to check whether they are in the same or different trend groups. The expression of the 6 miRNAs in the same group was evaluated in HCV infected Huh7 **(E)** and Huh7.5.1 cells **(F)**. The expression of the 20 miRNAs in the different group was also checked in HCV infected Huh7 **(G)** and Huh7.5.1 cells **(H)**.

Although global expression patterns were different between HCV-infected cell lines, we here tried to figure out HCV infection-specific miRNAs using further bioinformatic analyses. We performed series cluster using miRNA profiling data in Huh7 and Huh7.5.1 cell lines (**Figures 2A, B**). Comparing the significantly dysregulated miRNAs (False Discover Rate, FDR <0.01, fold change >2 or <0.5), we found that of the 125 (Huh7) and 77 (Huh7.5.1) dysregulated miRNAs identified, 38 miRNAs were overlapped and differentially dysregulated in both cell lines (**Figure 2C**), which we named them as HCV responsive miRNAs (**Figure 2D**). The expression changing trends of the overlapped dysregulated miRNAs were then compared, and only 6 of the HCV responsive miRNAs (miR-6079, miR-232a-5p, miR-4800-3p, miR-3202, miR-1245b-5p, miR-601) shared concordant expression changing trend across different cell lines (**Figure 2D**). The expression of the 6 concordant and 20 discordant miRNAs in HCV-infected Huh7 and Huh7.5.1 cell lines were verified using qPCR analysis (**Figures 2E–H**), consolidating our findings. The participation of these miRNAs in HCV infection and correlated hepatocellular carcinoma (HCC) progression were further clarified, and their potential targets were listed (**Table 1**).

**Table 1:** Information of miRNAs with indicated trend.

AccID	HCV participation	miRNApreName	HCC participation	Reported target genes	Trend
hsa-miR-6079	no	hsa-mir-6079	no	no	same
hsa-miR-323a-5p	no	hsa-mir-323a	no	MAPK9, CASP3, CASP6, CASP9, CCND1, CHAF1A, CD6, SMAD3, p73	same
hsa-miR-4800-3p	no	hsa-mir-4800	Biomarker of HCC prognosis	no	same
hsa-miR-3202	no	hsa-mir-3202-1/hsa-mir-3202-2	no	FAIM2, MeCP2	same
hsa-miR-1245b-5p	no	hsa-mir-1245b	no	TLR3, NKG2D, BRCA2	same
hsa-miR-601	no	hsa-mir-601	Tumor suppressor of HCC	NF-kappaB, ZEB1, KRT5, Cul3, HDAC6, PIK3R3, SIRT1, PTP4A1, B7-H3	same
hsa-miR-4423-3p	no	hsa-mir-4423	no	PIK3CA, SHC1, MYL3	different
hsa-miR-486-5p	no	hsa-mir-486-1/hsa-mir-486-2	Inhibition of HCC proliferation and metastasis	GSK3B, SULT2A1, PLAGL2, SMAD2, NRP-2, CBL, CD40, NEK2, H3F3B, IGF-1, NRF1, MARK1, Dock1, PIK3R1, CADM1, MAGI1, RASSF5, FOXO1, PTEN, TAK1, GAB2, CDK4, fibrillin-1, pim-1, OLFM4, ARHGAP5	different
hsa-miR-7156-5p	no	hsa-mir-7156	no	no	different
hsa-miR-520d-3p	no	hsa-mir-520d	Promotion of HCC development	SIRP alpha, AKT1, EphA2, SKA2, STAT3	different
hsa-miR-5009-3p	no	hsa-mir-5009	no	no	different
hsa-miR-5702	no	hsa-mir-5702	no	ZEB1, LUCAT1	different
hsa-miR-516a-5p	no	hsa-mir-516a-1/hsa-mir-516a-2	Promotion of HCC development, biomarker of HCC prognosis	MTA2, PHLPP2, FBXL18, MAP3K2, MAP2K4, MAPK10, MAPK11, TRAF6, HIST3H2A, MTHFR, MMP-2, TIMP-1, KLK10	different
hsa-miR-626	no	hsa-mir-626	Promotion of HCC proliferation and metastasis	LIFR, DKK3, Keap1, EYA4	different
hsa-miR-1290	no	hsa-mir-1290	Biomarker of HCC relapse	SOCS4, EMP2, MYLK, Napsin A, CCNG2, NKD1, IKK1, IRF2, INPP4B, NFIX, HIF3A, IGFBP3, HOXA1, FOXA1, GSK3β, NAT1, BCL2, KIF13B	different
hsa-miR-4789-5p	no	hsa-mir-4789	no	no	different
hsa-miR-337-5p	no	hsa-mir-337	Inhibition of HCC development	TCF7, JAK2, STAT3, Nrf-2, HMGA2, PTEN, HOXB7, KRAS	different
hsa-miR-4999-5p	no	hsa-mir-4999	no	PRKAA2	different
hsa-miR-486-3p	no	hsa-mir-486-1/hsa-mir-486-2	Biomarker of HCC relapse, mediation of sorafenib resistance	MAF, XRCC1, CYP1A1, UGT1A, HCN4, TLR4, NF-κB2, ECM1, FGFR4, EGFR, FOXP4, MGMT, FLNA, DDR1, ARID1B, SIRT2, BCL11A	different
hsa-miR-4634	no	hsa-mir-4634	no	no	different
hsa-miR-1248	no	hsa-mir-1248	no	BRCA1, TYMS, C3A, TRIM24, circHN1, RHCG, CITED2, ITPR3, AGTR1, IL-5	different
hsa-miR-6849-5p	no	hsa-mir-6849	no	no	different
hsa-miR-4516	no	hsa-mir-4516	Promotion of HCC progression	UBE2N, STAT3, PTEN, OTX1, MAPK10, PrPC, FOSL1, PTPN14, PVRL1, FN1, ITGA9, RPL37	different
hsa-miR-4703-5p	no	hsa-mir-4703	no	no	different
hsa-miR-3659	no	hsa-mir-3659	no	no	different
hsa-miR-4532	no	hsa-mir-4532	Biomarker of HCC	KCNJ11, LDOC1, SIRT6, HIC-1	different

**Figure 3:**
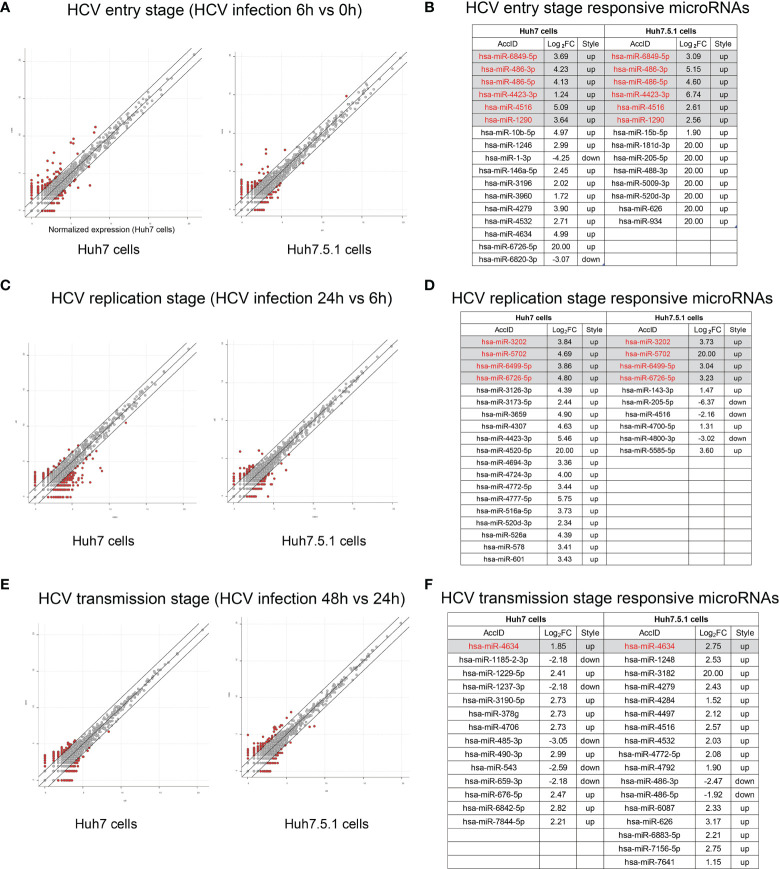
Responsive miRNA expressions during different stages of HCV infection. **(A)** The scatter plot showed the differentially expressed miRNAs during entry stage, and miRNAs with more than 2 folds of expression fold change (FC) above were listed with the overlapped 6 miRNAs indicated under the gray background **(B)**. Similarly, scatter plot indicating HCV replication stage was shown **(C)** and relevant responsive miRNAs were listed as well **(D)**. MiRNAs altered during HCV transmission were scatter plotted **(E)** and illustrated **(F)**.

We then compared the miRNA expressions during different stages of HCV infection. The entry stage of the differentially expressed miRNAs was defined as 6 h versus 0 h (**Figure 3A**). The altered miRNAs in Huh7 and Huh7.5.1 were analyzed, and 6 miRNAs were expressed concordantly (**Figure 3B**). Similarly, HCV replication stage-specific miRNAs were sorted out by 24 h versus 6 h to remove the impact of the previous stage in both cell lines (**Figure 3C**). Among them, 4 miRNAs overlapped between the two cell lines with the same alteration trend (**Figure 3D**). Finally, we compared miRNAs expressions between the 48 h and 24 h groups, and acquired transmission stage-specific miRNAs (48 h versus 24 h) (**Figure 3E**). In this stage, only miR-4634 showed concordant expression change in both Huh7 and Huh7.5.1 cells (**Figure 3F**). Of all these stage-specific miRNAs, none were repeatedly found, suggesting that the miRNA expression responding to the potential stimulus during the specific periods of HCV life cycle had their unique characters. Interestingly, all overlapped dysregulated miRNAs screened out from the three stages belonged to the upregulated style, probably reflecting the responsive state of the host body repressing or promoting viral infection.

### Cell Line Specific MiRNA Analysis Predicts Host Immune Responsive MiRNAs

**Figure 4:**
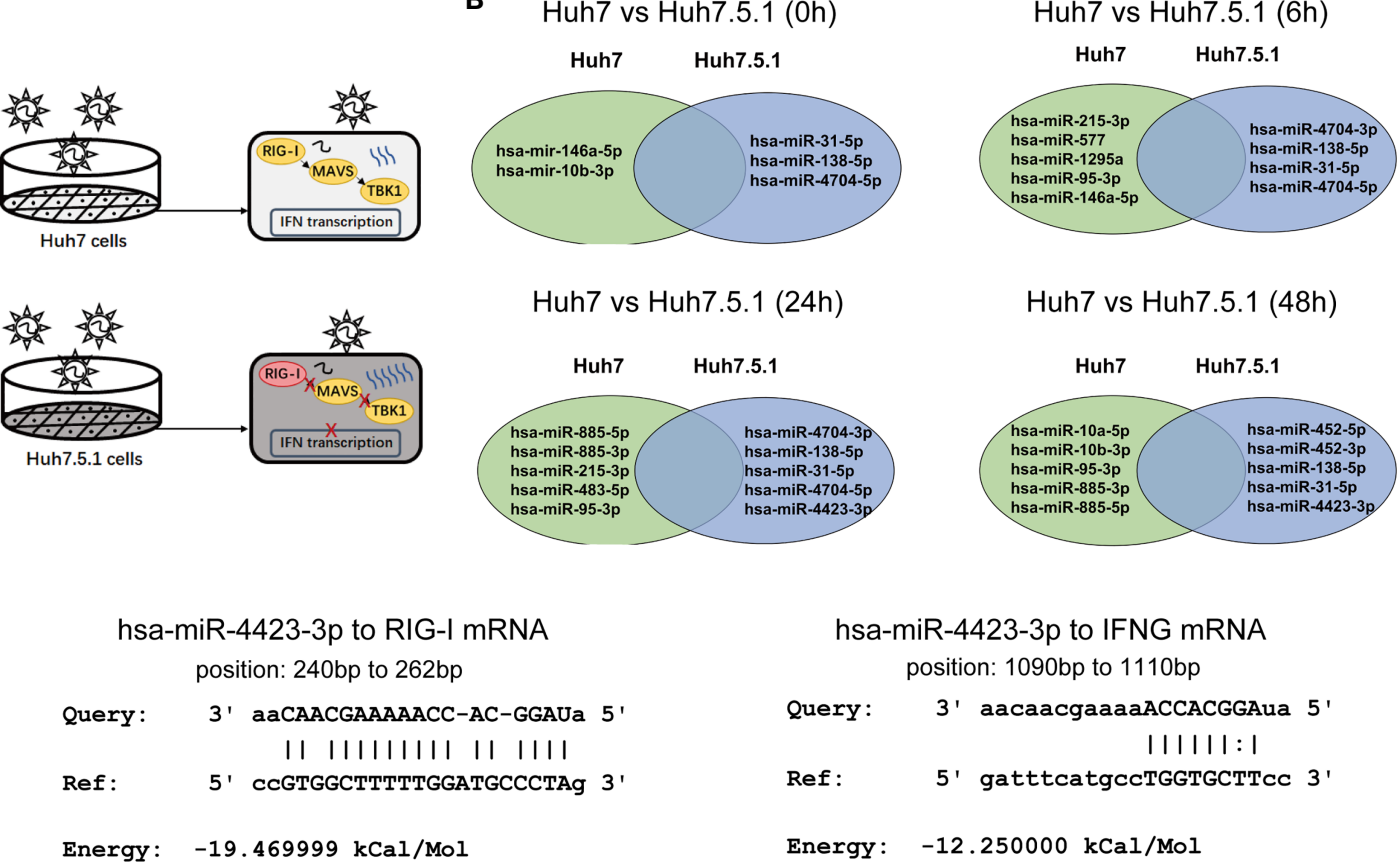
Candidate miRNA concerning IFN production pathway was selected from the differentially expressed miRNAs. **(A)** Diagram depicted the different responses between Huh7 and Huh7.5.1 cells after HCV infection. **(B)** MiRNAs with different change trends in the two cell lines, and the differentially expressed miRNAs were shown at respective infected periods (0, 6, 24, and 48 h). **(C)** Potential targets of miR-4423-3p were predicted and their interactions were illustrated. Results were shown as relative mRNA expression level.

Huh7.5.1 cells are derived from human hepatoma cell lines with a point mutation in the RIG-I gene, resulting in the suppression of host innate immune responses especially the reduction of type I interferon release to viral RNA (Rosen, 2013). Therefore, the expression pattern of dysregulated miRNAs in Huh7.5.1 cells might differ from the one in Huh7 cells during HCV infection, particularly with the miRNAs relating RIG-I/MAVS pathway (**Figure 4A**). We analyzed the stage-specific dysregulated miRNAs with distinct expression trends in the two cell lines, and found that several miRNAs (miR-4704-5p, miR-4704-3p, miR-885-5p, miR-885-3p, miR-138-5p, miR-31-5p, miR-4423-3p, miR-95-3p, miR-10b-3p, and miR-215-3p) showed similar expression change during various infection stages (**Figure 4B**). Here we predicted the binding potential of these candidates to the RIG-I/MVAS/IFNG pathway using the miRanda algorithm and found that miR-4423-3p had the potential to bind both the mRNAs of RIG-I or IFN-γ (**Figure 4C**). Thus, we proposed that miR-4423-3p might affect the expression of these two factors to regulate HCV infection.

### Functional Analysis of miR-4423-3p During HCV Infection

**Figure 5:**
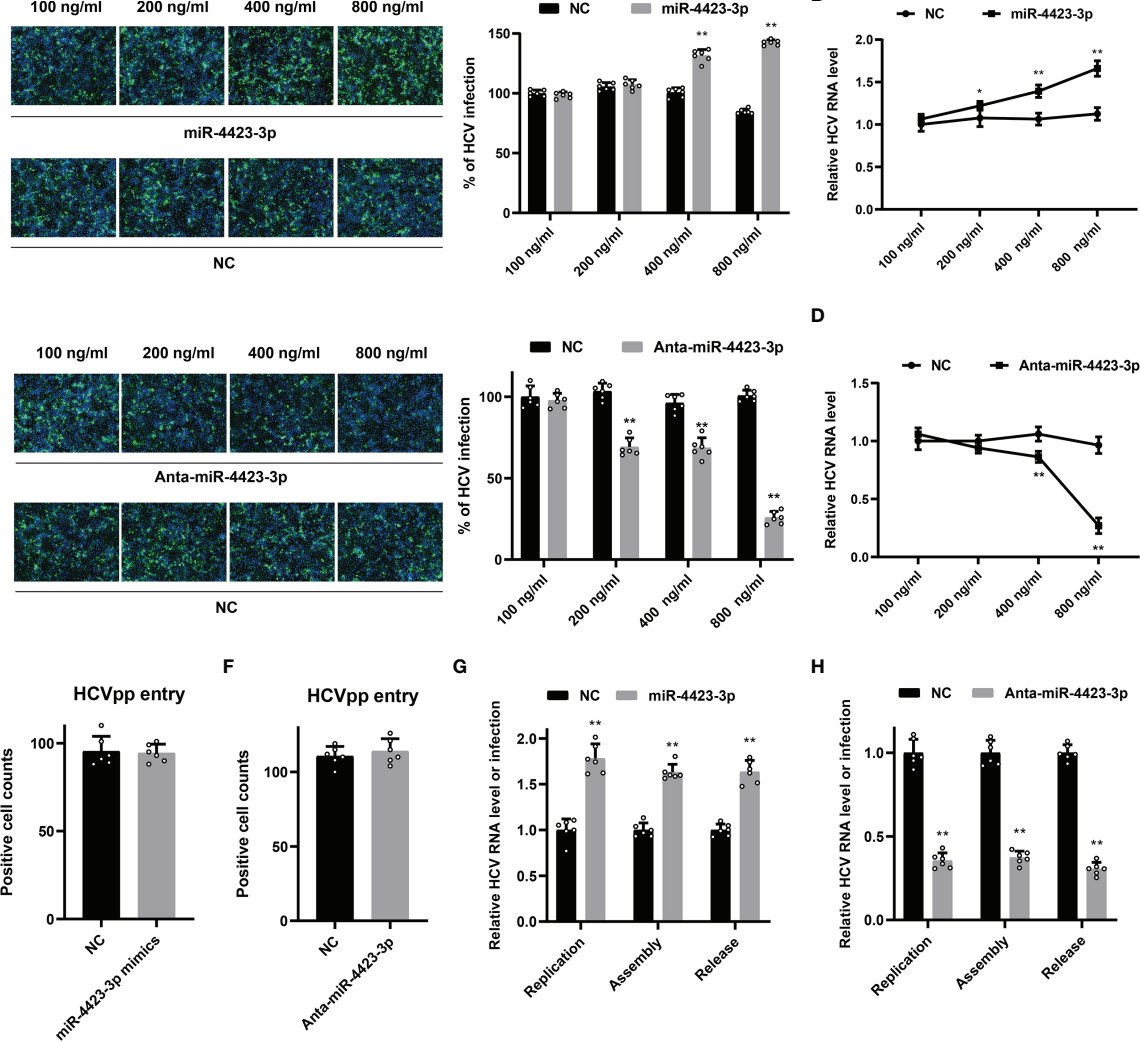
Function of miR-4423-3p on HCV infection. **(A, B)** Huh7 cells were transfected with indicated concentrations of miR-4423-3p mimics or scramble control and infected with HCVcc (MOI = 0.1) for 6h. Approximately 48 h after infection, HCV infection was analyzed by IF **(A)** and RT-qPCR **(B)**. **(C, D)** Huh7 cells were treated with various concentrations of miR-4423-3p inhibitors or scramble control, followed by infection of HCVcc (MOI = 0.1). Viral infection was also detected by IF **(C)** and RT-qPCR **(D)**. Results were shown as % of HCV infection or relative HCV RNA level. **p < 0.01 compared with negative control. **(E, F)** Huh7 cells were transfected with miR-4423-3p mimics **(E)** or inhibitors **(F)** and incubated with HCVpp for 6h. HCV entry efficiency was analyzed by counting the positive cells in respective wells. **(G, H)** Huh7 cells were treated with miR-4423-3p mimics **(G)** or inhibitors **(H)** for 24 h, and then transfected with HCV RNA. Aapproximately 24 h after transfection, parts of the cells were lysed to test intracellular HCV RNA level to evaluate viral replication. Parts of the cells were subjected to three cycles of freeze and thaw to test the intracellular viral infectivity to evaluate viral assembly. Cellular supernatant was collected to determine viral release. Results were shown as relative HCV RNA level or infection. *p < 0.05, **p < 0.01 compared with the negative control group.

To unveil the function of miR-4423-3p during HCV infection, Huh7 cells were transfected with the miRNA mimics and infected with cell culture-derived HCV particles (HCVcc) subsequently. The effect of miR-4423-3p was tested by both IF and qPCR (**Figures 5A, B**). As shown in **Figures 5A, B**, upregulation of miR-4423-3p promoted HCV infection at the transfected concentrations of 400 and 800 ng/ml, since both HCV core protein expression and viral RNA level were elevated potently. On the contrary, knockdown of miR-4423-3p by antagomir inhibited virus infection along with the elevated transfected concentrations, suppressing viral protein expression and reducing HCV RNA level (**Figures 5C, D**).

We then tried to clarify the fact at which step miR-4423-3p exerted its antiviral activity. HCV pseudoparticles (HCVpp) were utilized to evaluate the effect of miR-4423-3p on HCV entry. Huh7 cells transfected with either miRNA mimics or specific antagomirs were inoculated with HCVpp. Compared with the mock-treated group, both of the treatments failed to block HCVpp entry into the cells, indicating that miR-4423-3p did not affect virus infection during the entry step (**Figures 5E, F**).

Subsequently, the roles of miR-4423-3p were examined during the post-entry steps including viral replication, assembly, and release. HCV RNA was transfected into Huh7 cells by electroporation. Viral replication was analyzed by detecting intracellular HCV RNA levels. Viral assembly and release were evaluated by testing intracellular and extracellular virus infectivity respectively. As shown in **Figures 5G, H**, miR-4423-3p facilitated viral replication in HCV RNA-electroporated Huh7 cells, while downregulation potently reduced viral RNA levels. However, we did not notice the extra promotive or inhibitory effect of miR-4423-3p overexpression or downregulation on intracellular or extracellular virus infectivity since the alteration proportion of HCV infection was consistent with the ratio observed in replication during gain or loss of function experiments. Taken together, these results suggested that miR-4423-3p played an important role in HCV replication.

### MiR-4423-3p Regulated the Expression of RIG-I and Suppressed the Related Pathway in Targeted Cells

**Figure 6:**
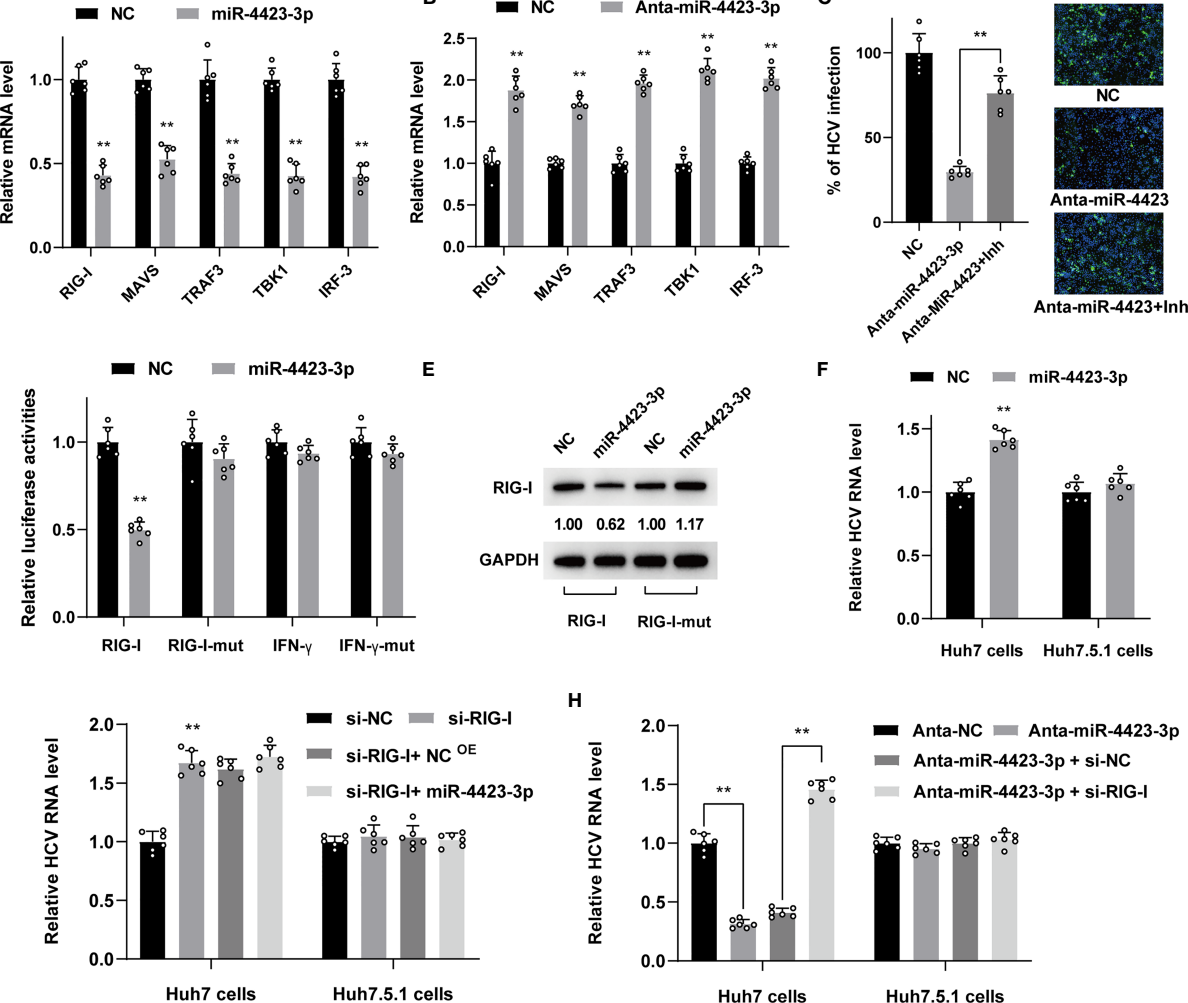
MiR-4423-3p affected viral infection through regulating the expression of RIG-I in Huh7 cells. **(A, B)** Huh7 cells were transfected with 800 ng/ml miR-4423-3p mimics **(A)** or inhibitors **(B)** together with scramble control and infected with HCVcc (MOI = 0.1). Cells were lysed to test the mRNA level of factor in RIG-I pathway 24 h after infection by RT-qPCR. **(C)** Huh7 cells were transfected with miR-4423-3p inhibitor (800 ng/ml) or scramble control and infected with HCVcc (MOI = 0.1). Parts of the cells were treated with TBK1 inhibitor of amlexanox. Viral infection was evaluated by IF. **p < 0.01 compared with the group without amlexanox treatment. **(D)** Dual luciferase reporter assay evaluating the firefly luciferase activities of the plasmids after miR-4423-3p overexpression in Huh7 cells. **(E)** The protein expression of RIG-I or IFN-γ was also tested by western-blot. The images were normalized by GAPDH and quantified. **(F)** Huh7 or Huh7.5.1 cells were transfected with 800 ng/ml miR-4423-3p mimics or scramble control, followed by HCV infection (MOI = 0.1), and viral infection was determined by RT-qPCR. **(G)** RIG-I expression was interfered by relevant siRNA in Huh7 or Huh7.5.1 cells. MiR-4423-3p mimics (800 ng/ml) or scramble control were transfected into the cells subsequently to analyze their effect on HCV infection (MOI = 0.1). **(H)** Huh7 or Huh7.5.1 cells were treated with MiR-4423-3p inhibitor (800 ng/ml) or scramble control and treated with RIG-I siRNA before the cells were infected by HCVcc (MOI = 0.1). Viral infection was detected by RT-qPCR. Results were shown as relative mRNA or HCV RNA level. **p < 0.01 compared with negative control.

It is well known that HCV infection will trigger innate immunity of IFN production through RIG-I/MAVS/TBK1 pathway. In order to validate the regulatory effect of miR-4423-3p on RIG-I pathway, we tested the expression level of essential factors involved in this process. We found the upregulation of miR-4423-3p contributed to the suppression of mRNA level of RIG-I and of the subsequent MAVS, TRAF3, TBK1, and IRF-3. While knockdown of miR-4423-3p resulted in the enhanced expression of these factors (**Figures 6A, B**). Additionally, a TBK1 inhibitor called amlexanox was utilized to verify the function of miR-4423-3p. The inhibitory effect of miR-4423-3p knockdown was partially reversed by the treatment of amlexanox (**Figure 6C**), further demonstrating the suppressive function of miR-4423-3p on RIG-I/MAVS/TBK1 pathway.

To gain more insights into the potential role of miR-4423-3p on the target gene, we constructed pMir-Report plasmids with 3’UTR region of predicted miR-4423-3p binding sites of RIG-I and IFN-γ, and their mutated sequence for verification using a luciferase reporter assay. Huh7 cells were transfected with the plasmid and treated with miR-4423-3p mimics subsequently. As shown in **Figure 6D**, only the RIG-I mRNA level was suppressed evidently with the RIG-I-mut level unchanged, while the elevation of miR-4423-3p had no effect on IFN-γ mRNA level, suggesting that RIG-I was the target of miR-4423-3p. The RIG-I protein expression was consistent with the mRNA level under miR-4423-3p treatment (**Figure 6E**).

Besides, we compared the effect of miR-4423-3p on both Huh7 and Huh7.5.1 cells. Differed from the promotive effect on HCV infection observed in Huh7 cells, miR-4423-3p overexpression had no effect in Huh7.5.1 cells (**Figure 6F**). In order to further confirm that the unique effect of miR-4423-3p in Huh7 cells was acquired by suppressing RIG-I, we transfected Huh7 or Huh7.5.1 cells with small interfering RNA targeting RIG-I. As shown in **Figure 6G**, in Huh7 cells, downregulation of RIG-I promoted HCV infection, and the original antiviral effect of miR-4423-3p was not available in the absence of RIG-I. However, this phenomenon was not observed in Huh7.5.1 cells (**Figure 6G**). Similarly, the inhibitory effect of miR-4423-3p interference on viral infection was reversed by the inhibition of RIG-I expression in Huh7 cells instead of Huh7.5.1 cells (**Figure 6H**).

## Discussion

HCV infection is a complicated process enclosing multiple host and viral components to participate in. During this process, the host defense system and viral persistence are two sides counteracting with each other and determine the outcome of whether the infection will be eradicated completely or progress into a more persistent chronic condition (Chigbu et al., 2019). The host body realizes its defensive functions mainly through immune responses especially the innate immunity triggered by several pathways including type I and III IFN responses to combat and control the spread of the virus (Hiroishi et al., 2008). Identifying and deciphering the key elements that regulate or affect these processes are crucial to understanding and controlling HCV infection.

MiRNAs have been considered to regulate diverse aspects of biological or pathological processes. There are various researches concerning the roles of miRNAs during HCV infection. MiR-122 was the well-known miRNA that was demonstrated to facilitate viral replication by binding to the 5’-UTR of HCV genome, thus becoming a key therapeutic target for HCV infection (Marquez et al., 2010). Several miRNAs such as miR-29, miR-143, and miR-185 were found to be dysregulated during chronic hepatitis C infection and correlated with HCV-related fibrosis and HCC (Bandyopadhyay et al., 2011; Singaravelu et al., 2015; Loureiro et al., 2020). However, no studies have been conducted to unveil the underlying miRNA regulatory networks comprehensively during HCV infection at indicated periods, an interplay embracing numerous genetic factors. In this study, we used high-throughput sequencing technology to test the miRNA expression level in both Huh7 and Huh7.5.1 cells. The miRNA signatures expressed in corresponding cell lines during specific infection periods might play important roles in the onset or progression of HCV infection, thus providing novel targets for diagnostic tests or therapeutic interventions.

To investigate how these miRNAs exerted antiviral activity against HCV, we performed comparative bioinformatic analysis. By utilizing this method, the differentially expressed miRNAs in the sequencing analysis were classified according to the different infection stages and targeted cell types. The selected candidate miRNAs were further characterized as entry, replication, and transmission responsive to reflect the miRNAs features during different stages of HCV infection. The differentially expressed miRNAs between Huh7 and Huh7.5.1 cells were found to be critical to the host antiviral processes. The regulatory networks of these vital miRNAs uncovered the existing factors which could be intervened during the pathological processes of HCV infection.

Among the many regulated processes of innate immunity, RIG-I/MAVS/TBK1 pathway was further studied. RIG-I senses viral RNA and activates mitochondria antiviral signaling protein (MAVS) which subsequently phosphorylate IFN regulatory factor 3 to promote IFN production (Saito et al., 2008). This pathway is invalid in Huh7.5.1 cells due to a single point mutation in the RIG-I gene. Therefore, the functional miRNA in the RIG-I pathway was figured out from the dysregulated miRNAs we compared between Huh7 cells and Huh7.5.1 cells after HCV infection. MiR-4423-3p was selected from the distinctly expressed miRNAs, since it is elevated in Huh7.5.1 cells at both 24 h and 48 h post infection, and also among the stage responsive miRNAs, especially during the replication process. Intensive investigation confirmed the promotive effect of miR-4423-3p on viral replication by decreasing the expression of RIG-I and the resulting inhibition of the subsequent host factors in IFN activation pathway. However, whether the blockage effects of miR-4423-3p on the downstream signaling molecules expression depended on RIG-I suppression only or involved other mechanisms required further investigations in the future.

Nevertheless, the actual *in vivo* effect and detailed mechanism of these miRNAs and their targeting genes still need further validation and confirmation. Still, our study characterized the miRNome profiling in HCV-infected cells during different infection periods and identified the crucial miRNA regulating the key factor of the innate immunity during HCV infection and progression. Our findings will broaden the understanding of the disease, and provide solid molecular evidence for the development of diagnostic or therapeutic agents.

## Data Availability Statement

The microRNA sequencing data of HCV infected-Huh7 and Huh7.5.1 389 cells can be downloaded from GEO at https://www.ncbi.nlm.nih.gov/geo/ under the accession number of GSE156205. The processed data can be found in **Supplementary Material**.

## Author Contributions

XJQ and CX designed the research. XJQ and BAW performed the research. XJQ wrote the paper. CX and ZTQ contributed to the proof-reading of the paper. All authors listed have made a substantial, direct, and intellectual contribution to the work and approved it for publication.

## Funding

This research was supported by grants from the National Natural Science Foundation of China (82172470), the Rising-Star Program of Science and Technology Commission of Shanghai Municipality (20QA1409200), and the Shanghai Changzheng Hospital High-Quality Research Cultivating Project (2020YCGPZ-207).

## Conflict of Interest

The authors declare that the research was conducted in the absence of any commercial or financial relationships that could be construed as a potential conflict of interest.

## Publisher’s Note

All claims expressed in this article are solely those of the authors and do not necessarily represent those of their affiliated organizations, or those of the publisher, the editors and the reviewers. Any product that may be evaluated in this article, or claim that may be made by its manufacturer, is not guaranteed or endorsed by the publisher.
